# Effect of photo-thermal acceleration on in-office bleaching

**DOI:** 10.1007/s10266-021-00612-5

**Published:** 2021-05-15

**Authors:** Feng Qi, Masayuki Otsuki, Noriko Hiraishi, Takashi Hatayama, Chamari Lasindra Wijethunge, Junji Tagami

**Affiliations:** Cariology and Operative Dentistry, Graduate School of Medical and Dental Sciences, Tokyo Medical and Dental University, 1‐5‐45 Yushima, Bunkyo‐ku, Tokyo, 113‐8549 Japan

**Keywords:** Tooth bleaching, Photo irradiation, Temperature, Hydrogen peroxide, Tooth color

## Abstract

The purpose is to evaluate the effect of photo-thermal acceleration on in-office bleaching efficiency using a bleaching agent without photocatalysts in vitro*.* Artificially discolored bovine lower incisors were prepared, and the mixed in-office bleaching material contained hydrogen peroxide 23% was applied by following treatment for 10 min: high-(HI group) and low-intensity LED lights (LI group), oven at 38 °C (OV group), and room temperature at 23 °C (RT group). Color was measured before and after bleaching and color difference (∆E*) was calculated. The data were statistically analyzed using a two-way ANOVA and Tukey’s post hoc test. The temperature change (∆*T*) of applied bleaching agent in HI and LI groups was measured using a thermography and was analyzed using a T test. The bleaching procedures were repeated 6 times. Irradiation in the HI group resulted in the highest Δ*E*, followed by the LI group whose Δ*E* was significantly lower. Both irradiated modes exhibited higher Δ*E* compared to non-irradiated OV and RT groups which were not significantly different from each other*.* The average temperature rise of bleaching agents in HI and LI groups after 10 min irradiation was 15.00 °C and 11.80 °C, respectively. The effect of photo-thermal acceleration was proved for an in-office bleaching agent without photocatalysts in vitro.

## Introduction

Tooth bleaching is one of the most popular and conservative dental procedures to lighten discolored teeth, which can be accomplished in-office, at-home, or dentist-supervised night-guard bleaching and bleaching with over-the-counter (OTC) products[1, 2]. For in-office bleaching, high concentration hydrogen peroxide (25–38%) is usually chosen because of the advantages of reduced total treatment time [3] and greater potential for immediate results that may enhance patient satisfaction and motivation, and the exposure of soft tissue is also avoided due to dentist’s control [4]. Therefore, compared to the other two bleaching methods, the in-office bleaching technique which combines with the use of light sources is appreciated by the patient to obtain faster results [5, 6].

The bleaching mechanism is described as the process that hydrogen peroxide decomposes to produce free radicals which react with chromogens composed of pigmented dual-bond compounds and/or metallic compounds [7–9]. This kind of chromogen accumulates in the tooth (intrinsic) or on the tooth (extrinsic) [9]. After reacting, the colorless molecular structures are generated resulting in tooth bleaching [10]. Meanwhile, many influence factors such as the temperature, light, or energy sources, photocatalyst, pH, the concentration of hydrogen peroxide, and the number of bleaching times affect the bleaching effect [11, 12].

The theoretical advantages of the light sources are to increase the chemical reaction rate of the bleaching process by producing heat [13] or to enhance the bleaching efficacy of bleaching materials by light activation of photocatalysts [14, 15] and consequently, to reduce the duration of the bleaching session. Suyama et al. [16] concluded that hydrogen peroxide-based bleaching agent containing titanium oxide photocatalyst exhibited a higher bleaching effect on account of light absorption. These photocatalysts reacted with the light at a very specific wavelength and transferred a small fraction of the light energy to the peroxide gel to activate bleaching procedures [14]. In addition to photocatalysts, higher bleaching effects were also induced by a higher increase in temperature caused by irradiation from high-intensity lights or lasers [17, 18]. Some specific colorants which give bleaching materials a colored appearance such as carotene also increase the heat conversion owing to the absorption of light is easier[16, 19]. Accordingly, a lot of research has utilized light sources with various wavelengths and power intensity such as plasma arc, lasers, quartz tungsten halogen lights, and light-emitting diodes (LED) to increase the bleaching efficacy of in-office bleaching materials with photocatalysts or photoactive colorants [17, 18, 20].

Some light sources, like LED lights that are specifically designed for bleaching procedures, have different modes illuminated for discolored teeth. Moreover, different modes generally have various wavelengths and different power intensities, thereby producing potentially different thermal emissions in the bleaching process [21, 22]. Usumez et al. [23] reported that the temperature rise was less by LED light irradiation during the polymerization of resin cement. Similar results regarding the temperature rise were obtained when LED was used in the bleaching process [16]. However, the relationship between the bleaching effect and temperature rises of bleaching material without photocatalysts or colorants caused by different power intensities LED lights was not clear. There is only limited information offered by the literature. Therefore, it should be investigated further by assessing the risks/benefits of LED lights, avoiding pulp damage caused by excessive heating in pursuit of high bleaching efficiency, and providing information to meet the need of seeking suitable light irradiation.

Therefore, the purpose of this study was to evaluate the effect of photo-thermal acceleration on in-office bleaching efficiency using a bleaching agent without photocatalysts in vitro.

Null hypothesis: (1) The in-office bleaching efficiency is not affected by temperature rise induced by light activation. (2) There is no difference in the temperature rise between the light groups.

## Materials and methods

### Artificially discolored bovine tooth models

#### Specimen preparation

Specimens were prepared according to the method of previous literature [11, 24]. Extracted frozen bovine teeth were defrosted with running tap water at room temperature before the experiment. After carefully removing soft-tissue remnants attached to the teeth by a scalpel, labial enamel surfaces were polished using silicon carbide papers from #600- to #800-grit (Sankyorikagaku, Saitama, Japan) under water cooling until almost 1 mm enamel in thickness was left to obtain flat standardized enamel surface. Then, two enamel-dentin specimens of approximately 5 × 5 mm in size were obtained from one tooth by rotary diamond saw (Mini Lab-cutter MC-110, Maruto instrument, Tokyo, Japan). The pulpal dentin of each specimen was covered with dental wax (Utility wax, GC, Tokyo, Japan), and the specimen was embedded in a cylindrical acrylic tube with 7 mm in height and 10 mm in internal diameter by polymerization of dental self-curing acrylic resin (Unifast III, GC, Tokyo, Japan). After dental wax was removed, the dentin surface was irrigated with the 5% sodium hypochlorite solution (Wako pure chemical, Osaka, Japan) for 30 s to remove organic tissue remnants followed by complete washing, drying, and finally etching with 40% phosphoric acid (K-etchant gel, Kuraray Noritake Dental, Tokyo, Japan) for 10 s, to encourage stain uptake into the tubule system. Then, specimens were polished again with #1,000- and #1,200-grit silicon carbide paper to get a smooth and flat enamel surface.

#### Artificial staining

The specimens were immersed in the black tea solution extracted by putting two tea bags (Lipton yellow label tea bags, Uniliver Japan, Tokyo, Japan) in 50 ml boiling water for 5 min and then stored in an incubator for 7 days at 37 °C. The solution was stirred once every day to avoid the precipitation of the solution and changed on the fourth day.

Stained labial surface color was recorded by CIE L*a*b value with a colorimeter (Color-checker NR-11, Nippon Denshoku, Tokyo, Japan) after washing and drying for each specimen. Each color measurement was done three times and the average of three measurements was taken as the measured value. The specimens in which L* value showed between 50 and 65 were selected for the following experiment and randomly assigned into four groups of 12 specimens (*n* = 12) each to complete the bleaching process. Baseline L*, a*, and b* values were applied, and enamel surface images were captured with a digital camera.

### Tooth bleaching procedure

The components of bleaching material used in the present study are listed in Table 1. Whiteessence whitening Pro consists of a liquid with 35% H_2_O_2_ and a viscous gel which contains thickener and sodium hydrogen carbonate. After mixing for 60 s, the concentration of H_2_O_2_ becomes 23%. Mixed in-office bleaching material (Whiteessence whitening Pro, White Essence, Tokyo, Japan) was applied on the enamel surface according to the manufacturer’s instruction and the applied surface was treated by following procedures for 10 min for all groups:

**Table 1:** Bleaching material used in this study

Product (manufacture)	Composition
Whiteessence whitening Pro, (White Essence, Tokyo, Japan)	Syringe Thickener Sodium hydrogen carbonate Purified water Liquid 35% hydrogen peroxide

*HI group* Blue LED (WE light Class II, White Essence, Tokyo, Japan) was irradiated using “High” intensity mode with power density 55 mW/cm^2^.

*LI group* The same light was irradiated using “Low” intensity mode with power density of 45 mW/cm^2^.

*OV group* Specimens were placed in an oven at 38 °C without light irradiation.

*RT group* Specimens were left in darkness at room temperature 23 °C.

**Fig. 1:**
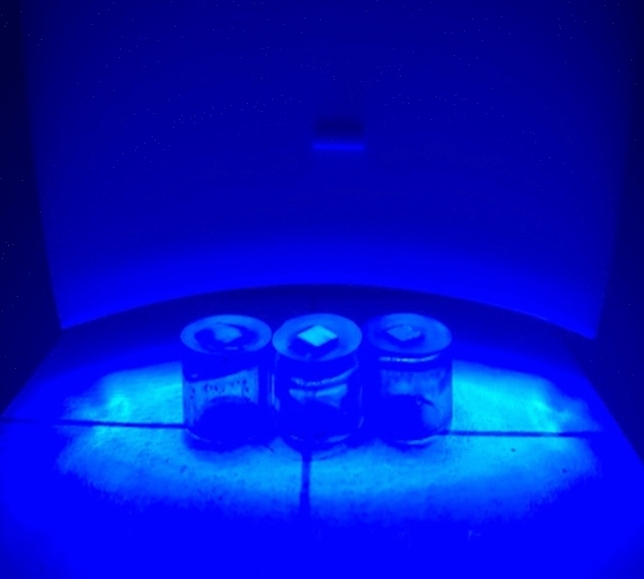
The multiple teeth covered with bleaching material were illuminated for 10 min at 5 mm from the LED

WE light Class II can be used to illuminate multiple teeth at the same time, and the distance between the blue LED and the specimen surface was fixed in 5 mm (Fig. 1). After 10 min application, the bleaching material was removed with damp cotton, and color measurements and photographs were performed again. The bleaching procedure and color measurements were repeated 6 times. Thus, the total time of bleaching was 60 min.

The color differences in ∆E* values obtained from L*, a*, and b* value at the baseline and after each bleaching application period were expressed by the formula:

$$\Delta E\, = \,((\Delta L*)^{{2}} \, + \,(\Delta a*)^{{2}} \, + \,(\Delta b*)^{{2}} )^{{{1}/{2}}}$$

ΔL*, Δa*, and Δb* represent the difference of L* values, a* values, and b* values between baseline and after bleaching respectively.

### Temperature measurement

Temperature measurement was carried out under controlled temperature (23 °C) condition using the air condition. In HI and LI groups, temperature change in ∆*T* of applied bleaching material was measured before and after light irradiation using a thermography (FLIR C2, FLIR System AB, Sweden). This is the first pocket-sized multifunction infrared thermography in the world, measuring a temperature range from – 10 °C to 150 °C. The mean of temperature change (∆*T*) at each bleaching step in the light irradiation groups is listed in Table 2.

**Table 2:** Mean of temperature change (∆*T*) at each bleaching step in HI and LI group

Bleaching time	HI group	LI group
1	14.80 (0.29)	11.60 (0.70)
2	15.00 (0.27)	11.70 (0.45)
3	15.10 (0.32)	11.80 (0.47)
4	15.10 (0.25)	11.80 (0.25)
5	15.10 (0.19)	12.00 (0.30)
6	14.90 (0.31)	12.10 (0.21)
Mean (SD)	15.00 (0.27)	11.80 (0.40)

### Statistical analysis

The assumption of normal distribution was tested using the Kolmogorov–Smirnov test. The color difference (ΔE*) was statistically analyzed using a two-way ANOVA and Tukey’s post hoc test (significance level 0.05) and a *T* test was used to identify differences in temperature change (∆*T*).

## Results

**Fig. 2:**
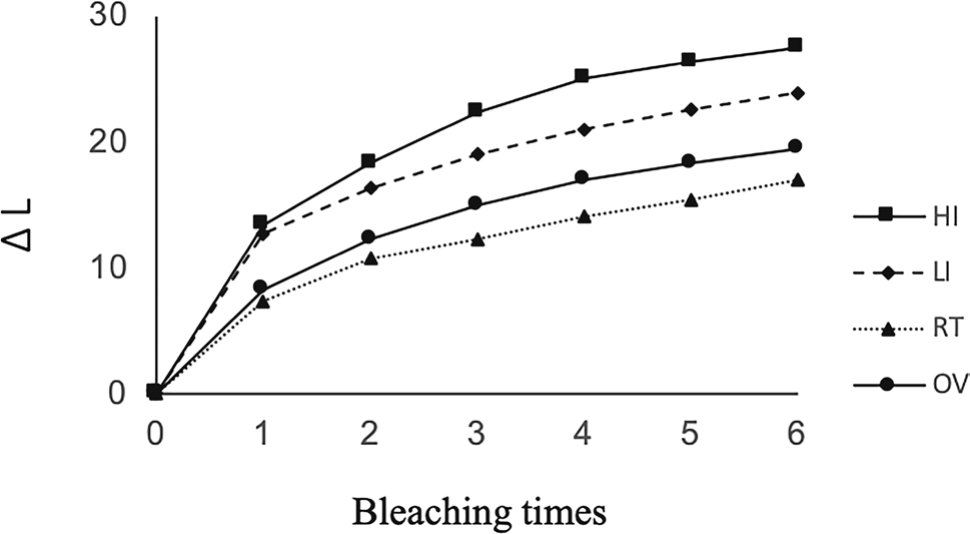
Mean of ∆L* at each bleaching step in all groups

**Fig. 3:**
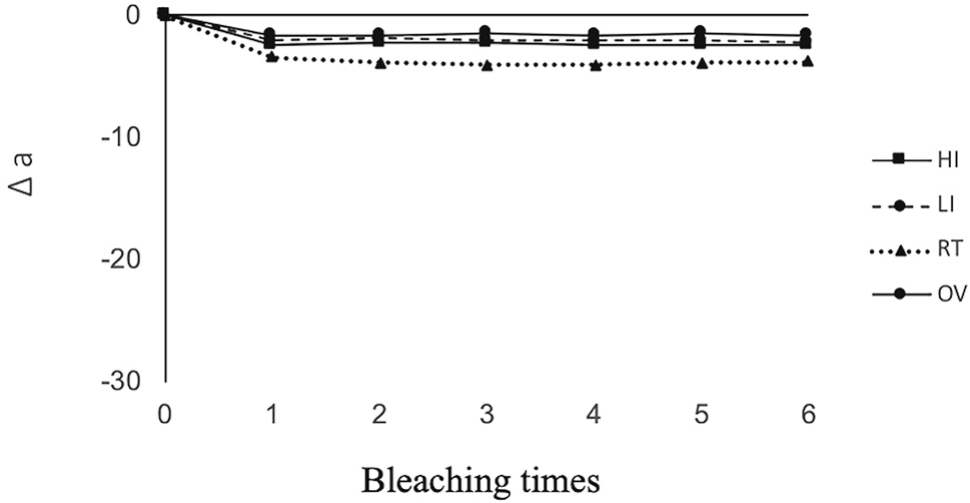
Mean of ∆a* at each bleaching step in all groups

**Fig. 4:**
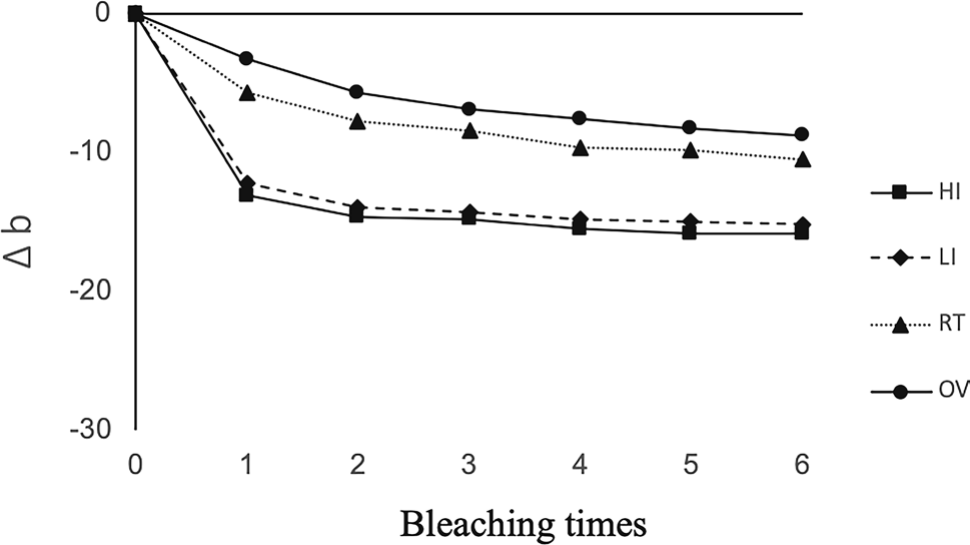
Mean of ∆b* at each bleaching step in all groups

**Fig. 5:**
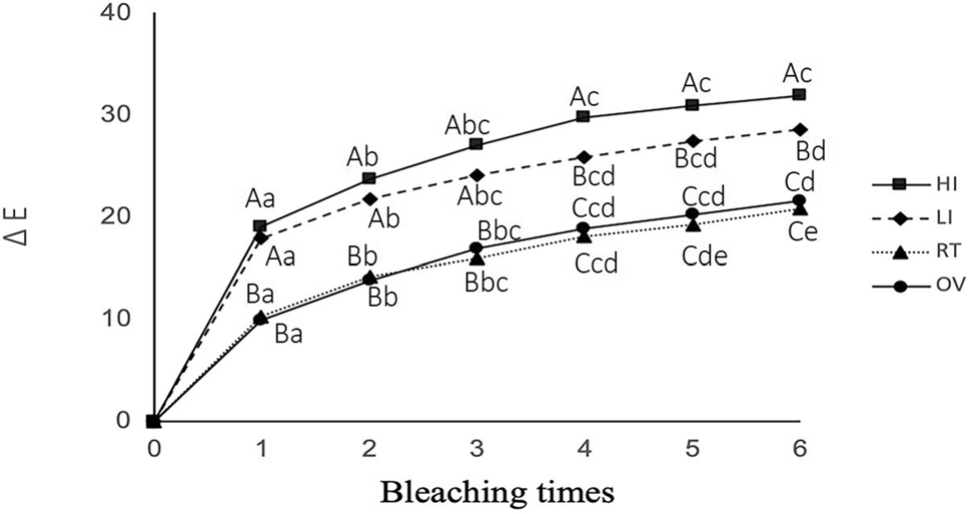
Mean of color difference values (∆E*) at each bleaching step in all groups. Different uppercase letters represent the statistical differences between groups and different lowercase letters represent the statistical difference between application times (*P* < 0.05).

**Fig. 6:**
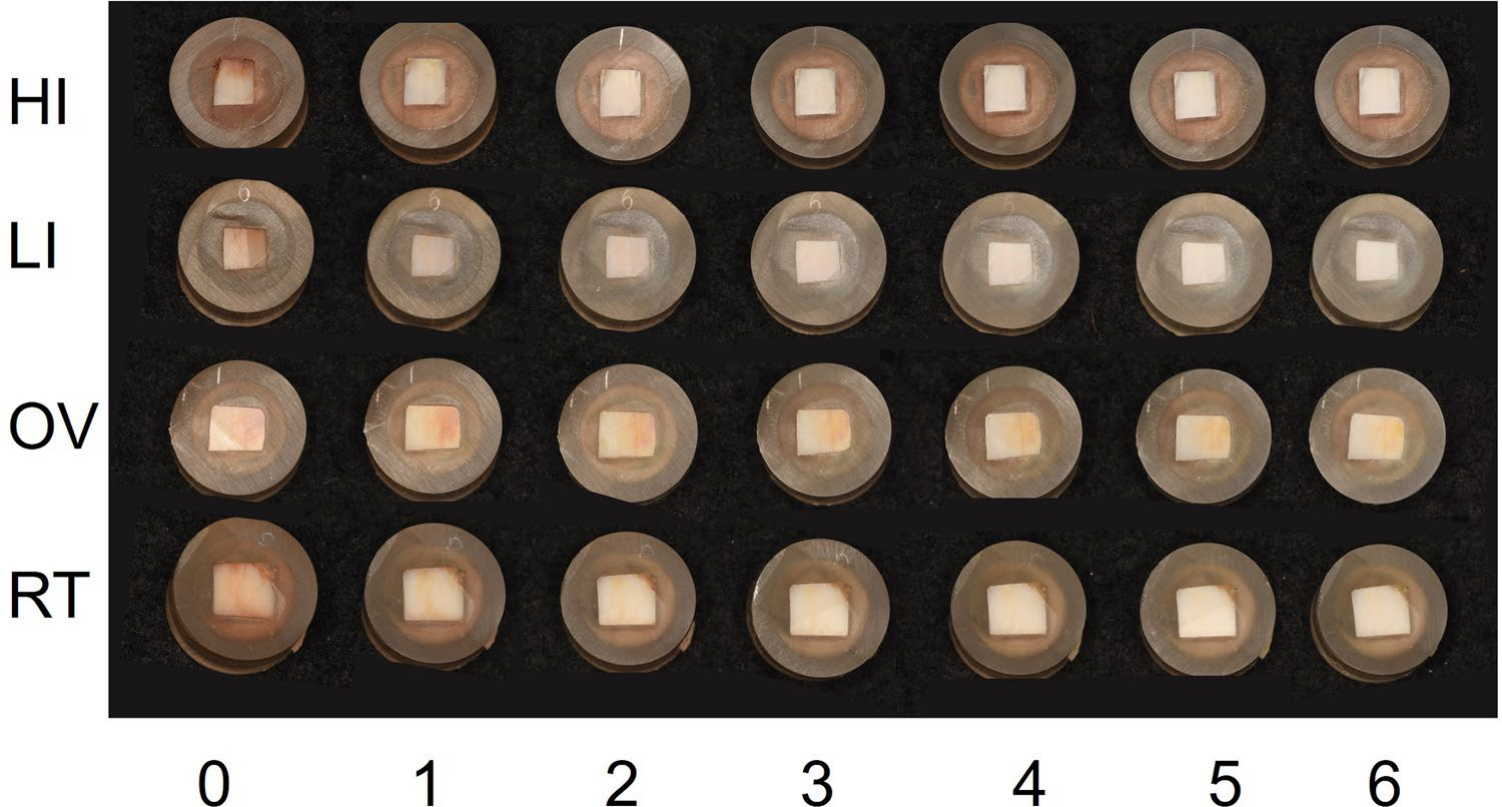
Typical color change in bovine teeth specimens of all groups at each bleaching step

The mean of ∆L*, ∆a*, ∆b*, and the color difference values (∆E*) at each bleaching step in all groups are displayed in Figs. 2, 3, 4 and 5, respectively. A positive bleaching effect in typical images of specimens is visually found in Fig. 6. ΔL* values gradually increased indicating the increase in brightness and Δb* values showed a decrease in all groups due to the blue transition of hue. However, the value of Δa* was little changed even after repeated bleaching in each group, indicating that it had little effect on the red–green change of teeth.

Color difference ΔE* values gradually increased with repeated bleaching in all groups. LED light in the high-intensity mode resulted in the highest ΔE* (from 19.04 for the first time bleaching to 31.86 for the sixth time bleaching), followed by low-intensity mode of the same light source whose ΔE* (from 17.89 to 28.47) was significantly lower (*P* < 0.05). Both light irradiation modes exhibited higher ΔE* compared to non-irradiated OV and RT groups (*P* < 0.05) which were not significantly different from each other (*P* > 0.05).

Two-way ANOVA showed that the number of bleaching agent application times and groups significantly affected Δ*E*. There was a significant interaction between the number of bleaching agent application times and the groups (*P* < 0.001).

**Fig. 7:**
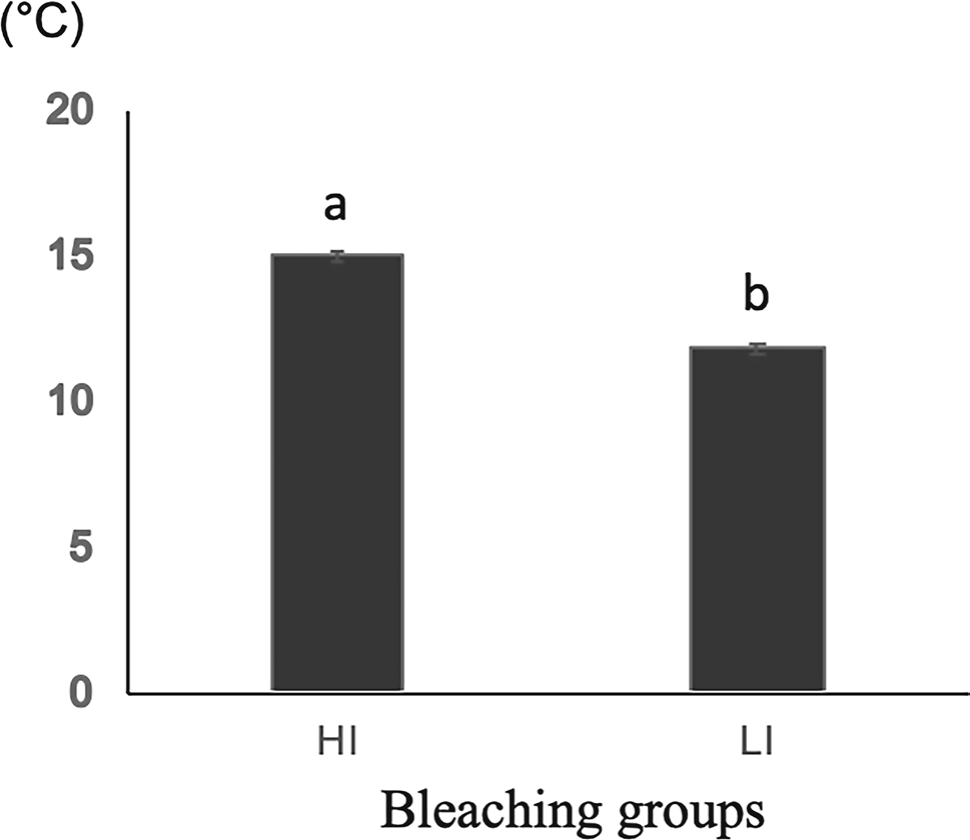
The average temperature change (∆*T*) of bleaching agent in HI and LI group after 10 min irradiation. Different lowercase letters represented significant differences between groups (*P* < 0.05).

According to the temperature measurement using the thermography, the temperature change in light irradiation groups was calculated. The mean of temperature change (∆*T*) at each bleaching step in the light irradiation groups is listed in Table 2. The temperature rise of bleaching agent in HI and LI groups after 10 min irradiation of each bleaching time was 15.00 ± 0.27 °C and 11.80 ± 0.40 °C (mean ± SD) respectively. HI group showed significant (*P* < 0.05) higher temperature rise in the bleaching process compared with the LI group (Fig. 7).

## Discussion

The efficacy of different light sources in the bleaching process has been extensively studied by many researchers. When the light is irradiated onto a bleaching product, respective photons are absorbed by bleaching agents, and its energy is converted into heat, which is the important factor for a temperature rise [18, 25, 26]. Then, redox reaction rate might be accelerated by rising temperature according to the molecular collision theory, and the likelihood of bonding cleavages and reactions is increasing due to molecules move faster and collisions among them become frequent [27]. In addition to it, more hydroxyl radicals that react with the organic molecules are produced from hydrogen peroxide when the temperature rises. It is known that a 10 °C increase in temperature doubles the production of hydroxyl radical [28]. As a result, many high-intensity light sources are used to increase the temperature of the bleaching material, thus enhancing the bleaching effect of hydrogen peroxide. However, some previous studies showed that the efficacy of light irradiation for bleaching materials was not obvious [29, 30]. The present study investigated the effects of the heating mechanism on tooth bleaching using blue light LED as, compared to heating without light, the use of bleaching material without photocatalysts eliminated the influence of light absorption by activating photocatalysts.

In vitro samples used in this method were stained in advance by black tea to simulate clinically discolored teeth. This approach has also been shown in many studies [11, 24]. As the most extensively used substitute for human teeth, bovine teeth eliminate the factors of insufficient quantity and bad quality because of different characteristics of human teeth like the degree of staining, mineralization status, or lesions which may cause a high deviation in the experimental results [31, 32]. Besides, it was reported that human and bovine teeth both exhibit similar physicochemical properties in hard tissues [33, 34]. Organic substances found in coffee, tea, and even wine are known to cause discolor on teeth. The stain made by black tea which is the typical extrinsic chromogens was easy to standardize, reproduce, and control [11] to simulate the discolor of teeth in vivo before the bleaching treatment.

In-office bleaching materials with neutral/alkaline pH are less aggressive to the enamel surface compared with acidic pH bleaching materials and maintain the pH during the application, allowing them to be left on the enamel surface for the whole application period [35]. In the present study, the bleaching material with a pH value of approximately 7.3 showed a greater bleaching effect. A dental colorimeter was used to measure the color change of teeth. ∆E value which was more than 3.3 was considered as a difference of color in the dental crown [14, 16, 36]. Compared with visual analysis, colorimeter breaks an overall color value into L*, a*, and b* components, and provides more efficient measurement in tooth color to make statistical analysis precise [37].

Our results suggested the higher the temperature rise when exposed to the visible light, the better the bleaching efficiency was obtained even without the presence of photocatalysts. The best bleaching effect occurred in the HI group, which produced a temperature rise of approximately 3 °C higher compared to the LI group. ΔE* also showed significantly higher in the irradiation group (HI and LI group) than the RT group which without any heat treatment. Therefore, the first null hypothesis that in-office bleaching efficiency is not affected by temperature rise induced by light activation was rejected. This finding agreed with the previous studies that high-intensity light produced more heat and had a positive effect on bleaching efficiency [14, 18, 19].

In the pilot study, the temperature of the bleaching agent reached 38 °C after 10 min high-intensity light irradiation. To compare the effect of the heat, the temperature in the OV group was set at 38 °C to achieve heat control. The use of an oven has not been found in the previous literature in tooth bleaching experiments; while it is difficult to interpret the result that a statistically significant difference was found between the light irradiation groups and the OV group. Although the potential roles of the oven in our study were to produce heat, it may happen that the heat generated by the oven reached the surface of the bleaching material through the air, making the temperature rise which was not high enough to affect the outcome of the bleaching procedure. However, compared with the heat produced by light irradiation, which was mostly concentrated in bleaching materials, the heat loss in the air might be relatively less. The dynamics of temperature change of bleaching materials might affect the bleaching effect in this study.

On the other hand, it could be thought that light itself may accelerate the reaction of hydrogen peroxide in the bleaching material. The peak wavelength of LED light in this study was 460 nm within the blue range, which indicated that it was more easily absorbed by the bleaching material due to higher scattering and penetrating [28]. As a result, more free reactive oxygen species might be produced from bleaching materials by visible-light irradiation. Moreover, De Moor et al. [38] reported that laser light can have several photochemical processes like photo-thermal effects (heat emission) or photooxidation (photobleaching) triggered by light absorption. These photochemical processes such as heat emission and photodynamic effects may also occur in LED lights, providing a greater effect on bleaching efficiency. Another factor that contributed to this result could be the photoreception of the pigment molecules in the dental surface. Because of the wavelength peak of light closed to the absorption peak of the molecules, light activation of the molecules made them breakdown into the smaller compound. The violet light LED has been proven to coincide with the absorption peak of pigmented molecules [10]. Further studies are necessary to evaluate the actual effect of the blue light LED on dental pigment molecules except for the effect of the light on the bleaching agent.

Although LED lights are proven to be effective in activating hydrogen peroxide, the adverse effects of heat production cannot be ignored. It was shown that a longer period of treatment not only speeded up penetration of peroxide into the pulp but also increased the temperature in intra-pulpal, sometimes caused irreversible damage so that limited clinical use [39]. For our in vitro study, the initial temperature of the bleaching material was 23 °C. In the light irradiation groups, the average temperature increase of the bleaching agent after 10 min irradiation was 15.00 °C in the high-intensity mode and 11.80 °C in the low-intensity mode to reach the final temperature of 38.00 °C and 34.80 °C, respectively. Therefore, the second hypothesis that there is no difference in the temperature rise between the light groups was rejected, because the HI group showed a significant (*P* < 0.05) higher temperature rise in the bleaching process compared with the LI group. Because of the insulating effect of the bleaching material reported by Sulieman et al., the presence of the bleaching gel could reduce the temperature increase in anterior teeth surface dramatically, by 87–96% [40]. Besides, the cooling effect of simulated pulp microcirculation in the thermal behavior of the dentine also has been proved in dentine pulp complex ex vivo [41]. It might occur the temperature rise in the pulp cavity is far less than the maximum temperature rises of the bleaching agent in this study. The lower limit of the temperature that threatened pulp vitality was 5.5 °C reported by Zach et al. [42]. Therefore, further studies about intra-pulpal temperature measurements are needed to evaluate the effect of temperature rise on bleaching agents. The use of the LED light containing different intensities in this study evaluated the extent of the temperature rise which is important to study the safe temperature level of the bleaching procedure.

Moreover, changes in enamel and dentin micromorphology may be occurred by the long contact time between the bleaching agent with different concentrations and pH and teeth surfaces, such as the presence of erosions and/or porosity [43] and changes in mineral content as well as in surface micro-hardness [43–45]. However, a few studies have focused on the effect of increasing temperature of bleaching agent on tooth enamel and dentin. Furthermore, hydrogen peroxide diffusion and changes in enamel and dentin micromorphology studies are necessary to evaluate the bleaching efficiency and tooth sensitivity caused by increasing temperature.

Despite the limitation of this in vitro study, the effect of photo-thermal acceleration was proved for an in-office bleaching agent without photocatalysts, not only the heat generated by light exposure but also light itself may result in more effective bleaching in tooth bleaching.
